# Adaptation for *Staphylococcus aureus* to hosts via insertion mutation in the accessory gene regulator *agrC* gene: decreased virulence and enhanced persistence capacity

**DOI:** 10.1128/spectrum.01497-24

**Published:** 2024-11-29

**Authors:** Jiawei Chen, Yun Wu, Ying Zhu, Li Zhang, Yingchun Xu, Yali Liu

**Affiliations:** 1 Department of Laboratory Medicine, State Key Laboratory of Complex Severe and Rare Diseases, Peking Union Medical College Hospital, Chinese Academy of Medical Sciences and Peking Union Medical College, Beijing, China; 2 Graduate School, Peking Union Medical College, Chinese Academy of Medical Sciences, Beijing, China; University of West London, London, United Kingdom

**Keywords:** *Staphylococcus aureus*, *agrC *gene, virulence, persistence

## Abstract

*Staphylococcus aureus* is an important human pathogen due to its vast array of virulence factors regulated by multiple regulatory mechanisms, including the accessory gene regulator. In this study, two *S*. *aureus* strains were simultaneously isolated from the blood of a febrile patient, belonging to the same clone, designated as 23H with a complete hemolytic phenotype, and 23B, exhibiting an incomplete hemolytic phenotype. The genomic comparison between strains 23B and 23H revealed that 23B had a single adenine base insertion at position 923 in the *agrC* gene, leading to a functional loss of the encoded AgrC. Experimental findings showed that strain 23B had decreased hemolytic activity, lower cytotoxicity against human alveolar epithelial A549 cells and in the *Galleria mellonella* model, and a reduced ability to survive intracellularly after infecting macrophages, in comparison to 23H. Conversely, 23B exhibited enhanced biofilm formation, greater adherence to A549 cells, and increased persistence in the face of vancomycin and daptomycin treatment. Transcriptomic analysis revealed that 23B upregulated surface protein-encoding genes while simultaneously reducing the expression levels of virulence factors, highlighting the intricate regulatory adjustments facilitating its persistence and reducing pathogenic potential. ATP assay results indicated that 23B maintained elevated ATP levels during the exponential phase yet exhibited reduced levels in the stationary phase when compared with 23H. Our findings suggested that the mutation in the *agrC* gene of *S. aureus* results in diminished virulence but markedly enhances persistence. This mutated strain warrants clinical attention because it may lead to treatment failures and persist in patients.

**IMPORTANCE:**

In clinical antimicrobial therapy, bacterial strains often develop resistance to antimicrobial agents. Additionally, mutations in their gene regulatory networks can increase their persistence, especially in immunocompromised patients. This study identified an insertion mutation in the accessory gene regulator, *agrC* gene, carried by a *Staphylococcus aureus* strain isolated from the blood of a febrile patient, leading to the functional loss of AgrC. Further research revealed that despite the reduced virulence of the mutated strain, it significantly bolstered the capacity to adapt and endure within the host during prolonged infections. This was evidenced by increased adhesion and biofilm formation capabilities, development of antimicrobial tolerance, and decreased ATP levels linked to persistence. Therefore, monitoring these mutations in *S. aureus* is crucial clinically, as they can complicate treatment strategies.

## INTRODUCTION

*Staphylococcus aureus* is a versatile human pathogen responsible for both hospital- and community-acquired infections, and it is capable of affecting numerous body sites including the skin, bloodstream, respiratory tract, heart, skeletal system, and areas surrounding implanted medical devices (1, 2). The ability of *S. aureus* to infect diverse organs and cause a variety of acute and chronic conditions stems from its extensive array of virulence factors (3). Key among these are its extracellular protein toxins, such as enterotoxins, toxic shock syndrome toxin 1 (TSST-1), exfoliative toxins (ETs), hemolysins, epidermal cell differentiation inhibitors (EDINs), and panton-valentine leukocidin (PVL), that significantly contribute to its pathogenicity (4).

*S. aureus* can modulate the expression of virulence factors through gene regulatory networks such as the two-component system and quorum-sensing regulators, enabling it to effectively respond to the challenges posed by environmental changes (5). The accessory gene regulator (*agr*) system, combining a classical two-component system and a quorum-sensing system (6), plays an important role in the regulation of virulence factors and metabolism genes (7). Structurally, the *agr* system is organized into two transcriptional units, RNAII and RNAIII, oriented in opposite directions and initiated by the P2 and P3 promoters, respectively (8). RNAII is an operon that encodes four genes (*agrBDCA*), within *agrB* and *agrD* genes are involved in quorum-sensing, while *agrC* and *agrA* genes constitute the two-component system. AgrD serves as a foundational precursor peptide, which is intricately processed by the membrane-associated protease AgrB to produce an autoinducing thiolactone peptide (AIP). The extracellular accumulation of AIP is detected by the membrane-bound kinase AgrC, which subsequently undergoes autophosphorylation within its intracellular histidine kinase domain. This phosphoryl group is then transferred to cognate response regulator AgrA, which, in turn, activates transcription of its own RNAII transcript and the regulatory RNAIII (9).

RNAIII not only directly encodes δ-hemolysin (Hld) but also acts as a multifaceted regulator. It can enhance the transcription of numerous extracellular protein genes, notably affecting the expression of virulence factors such as α-toxin (Hla), serine protease (SplA-F, SspA), cysteine proteases (ScpA, SspB), and leucocidins (LukAB, lukGH) (10–12). In contrast, the expression of certain surface proteins such as protein A (Spa), cell wall secretory protein (IsaA), and surface receptors (MnhA, MnhF, and MnhG) is downregulated by RNAIII (10, 11), which plays key roles in adhesion and in defense against the host immune system. Furthermore, the *agr* system regulates the production of phenol-soluble modulins (PSMs), which bind released phospholipids, thereby preventing daptomycin inactivation (7, 13). Consequently, inactivation of the *agr* system affects *S. aureus* traits such as strain adhesion, biofilm formation, and virulence phenotype. In this study, two strains of *S. aureus* with frameshift mutations in the *agrC* gene were isolated simultaneously from a clinical infection patient. We aim to explore the consequences of the non-functional AgrC on the pathogenicity of *S. aureus* in a clinical setting, thereby providing insights into its role in infection dynamics.

## RESULTS

### Phenotypic characterization of two *Staphylococcus aureus* strains

**Fig 1:**
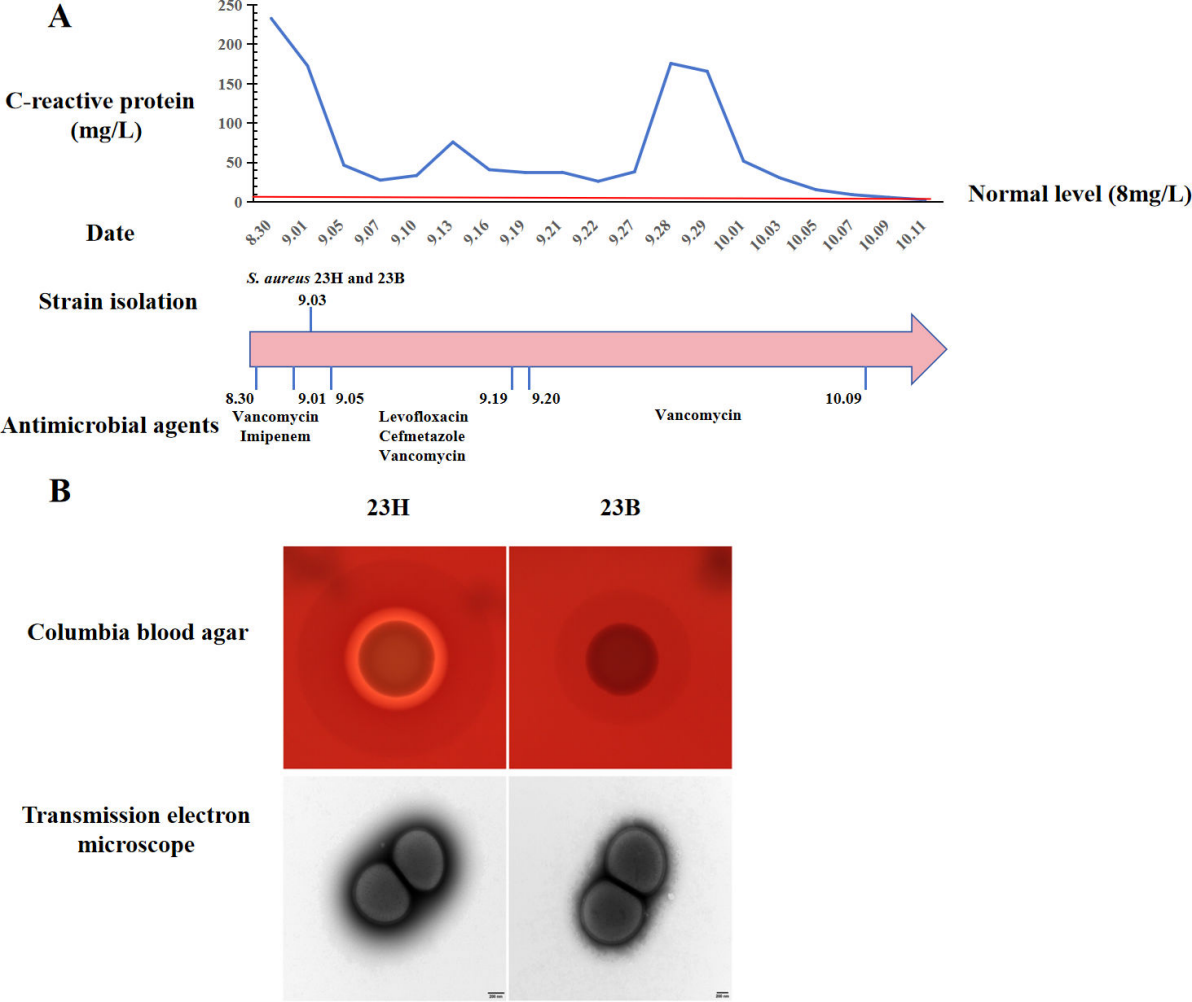
Case description of the infected patient and isolated *S. aureus* strains. (A) C-reactive protein levels, antimicrobial treatment process, and strain isolation timeline in an infected patient. (B) The morphological features of two *S*. *aureus* strains under transmission electron microscopy and in Columbia blood agar.

The *S. aureus* strains 23B and 23H exhibited nearly identical susceptibility profiles to antimicrobial agents, demonstrating susceptibility to almost all tested antimicrobial agents except for erythromycin and clindamycin (Table S1). These two strains showed a distinct hemolytic difference on Columbia blood agar, with strain 23H displaying a complete hemolytic phenotype, while strain 23B exhibited an incomplete hemolytic phenotype (Fig. 1B). Observation under transmission electron microscopy (TEM) revealed that strain 23B exhibited a rougher edge accompanied by irregularities, which were evident as protrusions, indentations, or uneven contours along the cell membrane. Conversely, strain 23H exhibited a smoother surface morphology, characterized by a more uniform appearance (Fig. 1B). Additionally, both *S. aureus* displayed comparable growth curves under standard condition at 37℃ in BHI (data not shown).

### Whole-genome analysis

The results of whole-genome analysis revealed both *S. aureus* strains 23B and 23H belonged to the sequence type 59 (ST59) and spa type t163. Furthermore, analysis of the antimicrobial resistome and toxome profiles demonstrated the presence of two antimicrobial resistance genes, *blaZ* and *ermC*, alongside a diverse repertoire of virulence-related genes encompassing adherence factors, enzymes, immune evasion mechanisms, secretion systems, and toxins. Comparative genomic analysis between strains 23H and 23B revealed that strain 23B exhibited a frameshift mutation characterized by an insertion of an A base at position 923 within the *agrC* gene (Table S2), indicating that they were the same clone according to the previous definition (14). This mutation led to the premature termination of the codon, resulting in truncation of the AgrC protein to only 71% of its normal amino acid sequence.

### Virulence assays

**Fig 2:**
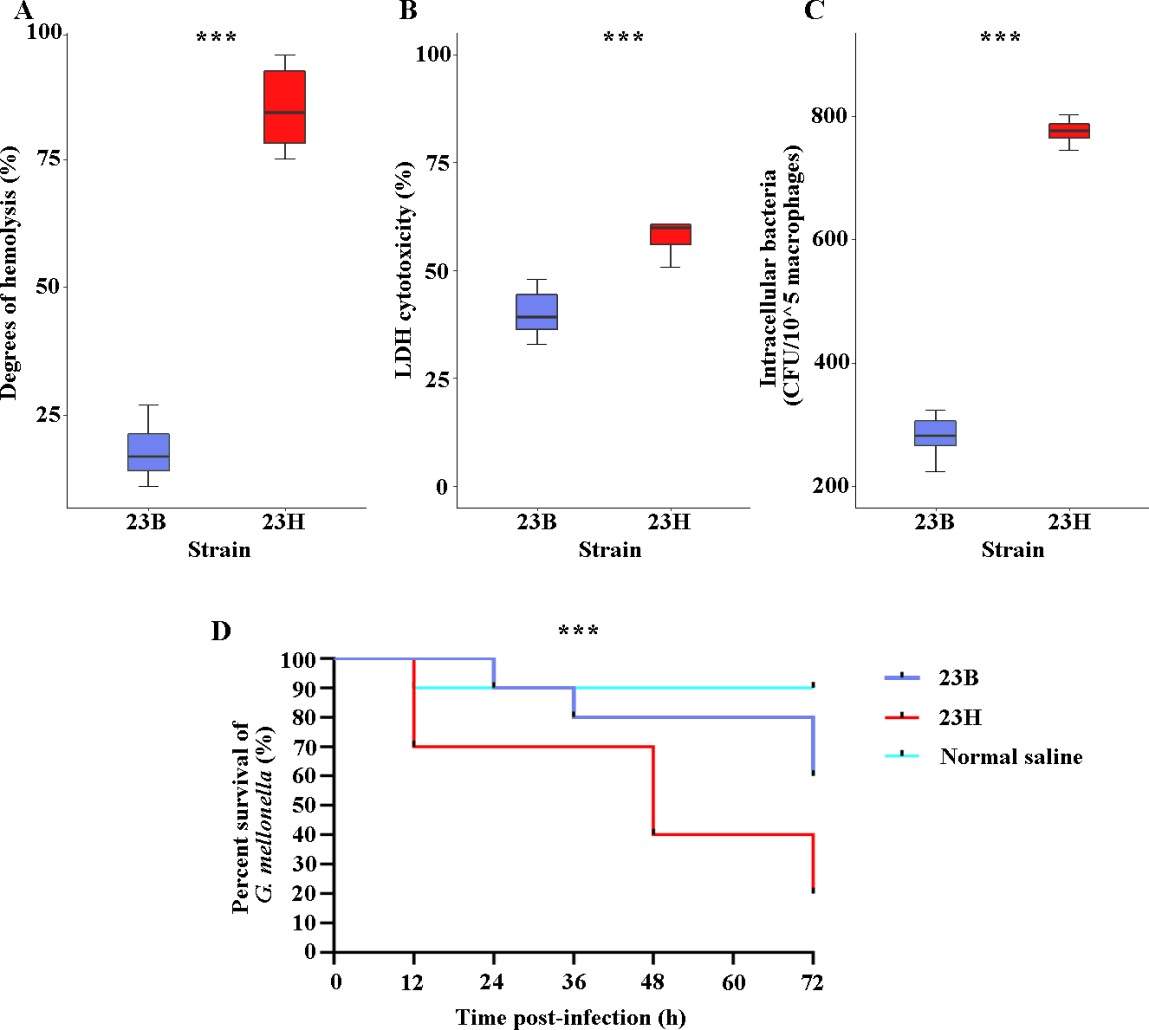
Comprehensive analysis of virulence assays in *S. aureus* strains 23B and 23H. *** represented a *P*-value of <0.001. (A) The degree of hemolysis of rabbit blood cells of strains 23B and 23H. (B) The LDH cytotoxicity rate of 23B and 23H on A549 epithelial cells. (C) The macrophage survival assay of strains 23B and 23H. (D) The survival curves of *G. mellonella* after infection of strains 23B and 23H.

Given the association of the *agrC* gene of *S. aureus* with virulence (7)**,** we conducted several experiments to assess virulence in strains 23B and 23H. Initially, we compared their hemolytic capacity, and consistent with the morphology observed on blood agar plates (Fig. 1B), strain 23H demonstrated significantly stronger hemolytic ability compared to strain 23B (85.0% vs 17.9%, *P* < 0.01) (Fig. 2A), potentially indicating a higher virulence level in strain 23H. Subsequently, more detailed experiments were undertaken to compare the virulence phenotypes of the two strains. The LDH release assay revealed that strain 23H exerted a stronger cytotoxic effect on A549 cells (57.82% vs 40.08%, *P* < 0.01) (Fig. 2B). The hypervirulent phenotype of both strains was also evident in the *Galleria mellonella* infection model. The survival rates of *G. mellonella* injected with 23H at 12 h, 48 h, and 72 h post-infection were 70%, 40%, and 20%, respectively, significantly lower than those infected with the strain 23B (Fig. 2D). Moreover, the capacity to resist macrophage phagocytosis is another manifestation of bacterial virulence (15). Over a 4 h period, significantly fewer 23B isolates were internalized by mouse macrophages compared to 23H (281 CFU vs 775 CFU, *P* < 0.01) (Fig. 2C). These findings compellingly suggest that 23H exhibits a more potent virulence phenotype than 23B.

### The persister, adhesion, and biofilm formation assays

**Fig 3:**
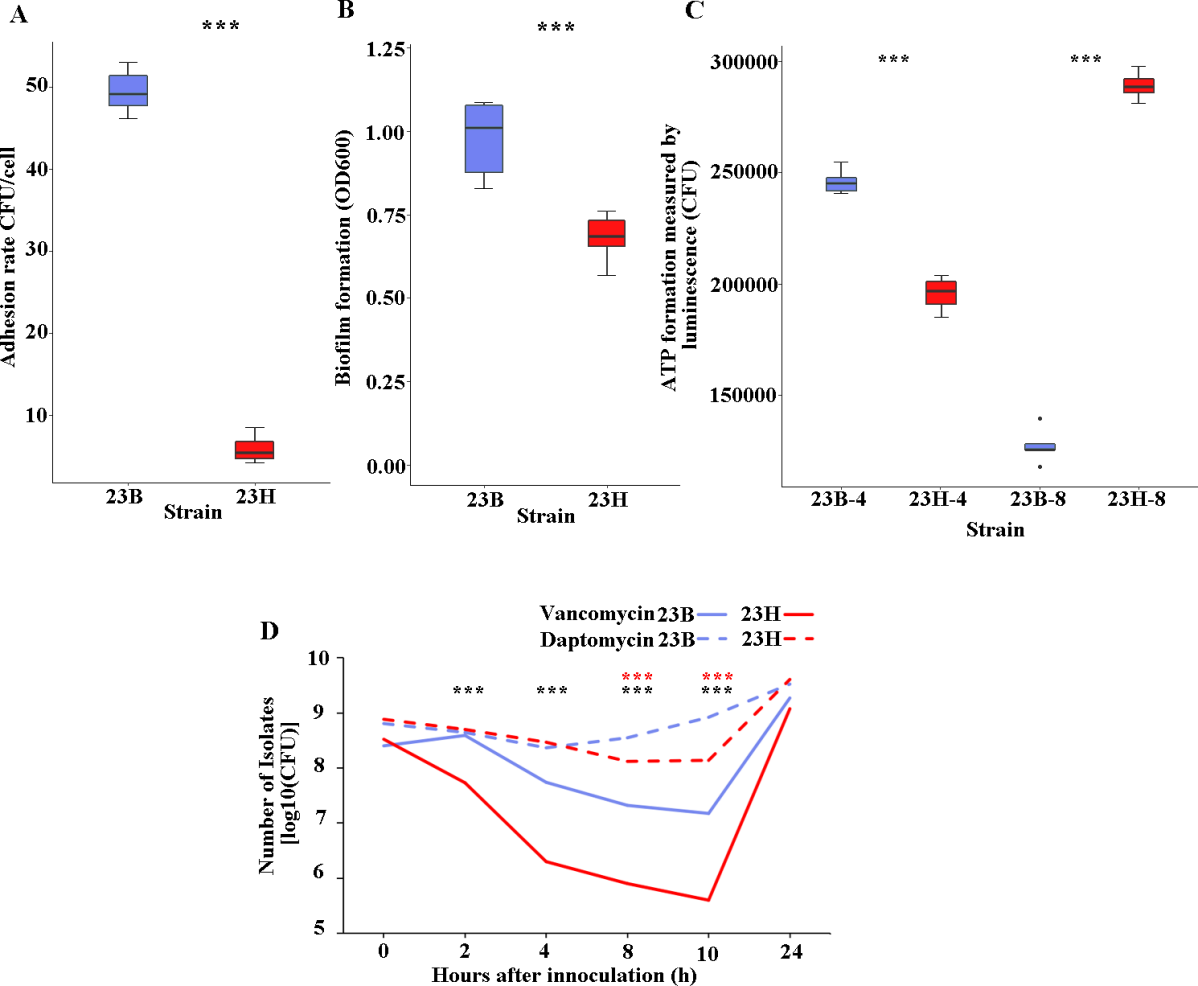
Comprehensive analysis of the persistence capacity of *S. aureus* strains 23B and 23H. *** represented a *P*-value of <0.001. (A) Quantification of adhesion and internalized colony counts of strains 23B and 23H in A549 epithelial cells. (B) Biofilm formation by strains 23B and 23H after 48 h of growth. (C) The ATP levels in exponential and stationary phases of the populations of strains 23B and 23H. 23B-4 and 23H-4 represented strains 23B and 23H during the exponential phase at 4 h and 23B-8 and 23H-8 represented strains 23B and 23H during the stationary phase at 8 h. (D) Daptomycin and vancomycin time-killing profile of strains 23B and 23H. The *** of red represented the *P*-value of daptomycin, and the *** of black represented the *P*-value of vancomycin.

The capacities for persistence, adhesion, and aggregation of *S. aureus* are fundamentally vital for its effective colonization, infection, and dissemination among humans (16). In this study, the A549 cell line was employed as a model to assess the adhesion abilities of strains 23H and 23B. A significantly increased adhesion of strain 23B was observed compared to that of strain 23H after 3 h incubation (48.5 vs 4.5 CFU/cell, *P* < 0.01) (Fig. 3A). Moreover, strain 23B demonstrated enhanced biofilm formation relative to strain 23H (Fig. 3B), suggesting that strain 23B may lead to more severe intercellular bacterial aggregation. Additionally, we evaluated the time-kill curves of daptomycin, levofloxacin, vancomycin, oxacillin, or gentamicin against these two strains. The results showed that, with the exception of daptomycin and vancomycin, the remaining antimicrobial agents showed no significant differences in impacting the survival of the strains. Notably, at intervals of 2, 4, 8, and 10 h exposed to vancomycin, the survival ability of strain 23B was significantly higher than that of strain 23H (*P* < 0.01). Similarly, strain 23B demonstrated enhanced survival at 8 and 10 h under the effect of daptomycin compared to strain 23H (*P* < 0.01) (Fig. 3D).

### Transcriptome result analysis

**Fig 4:**
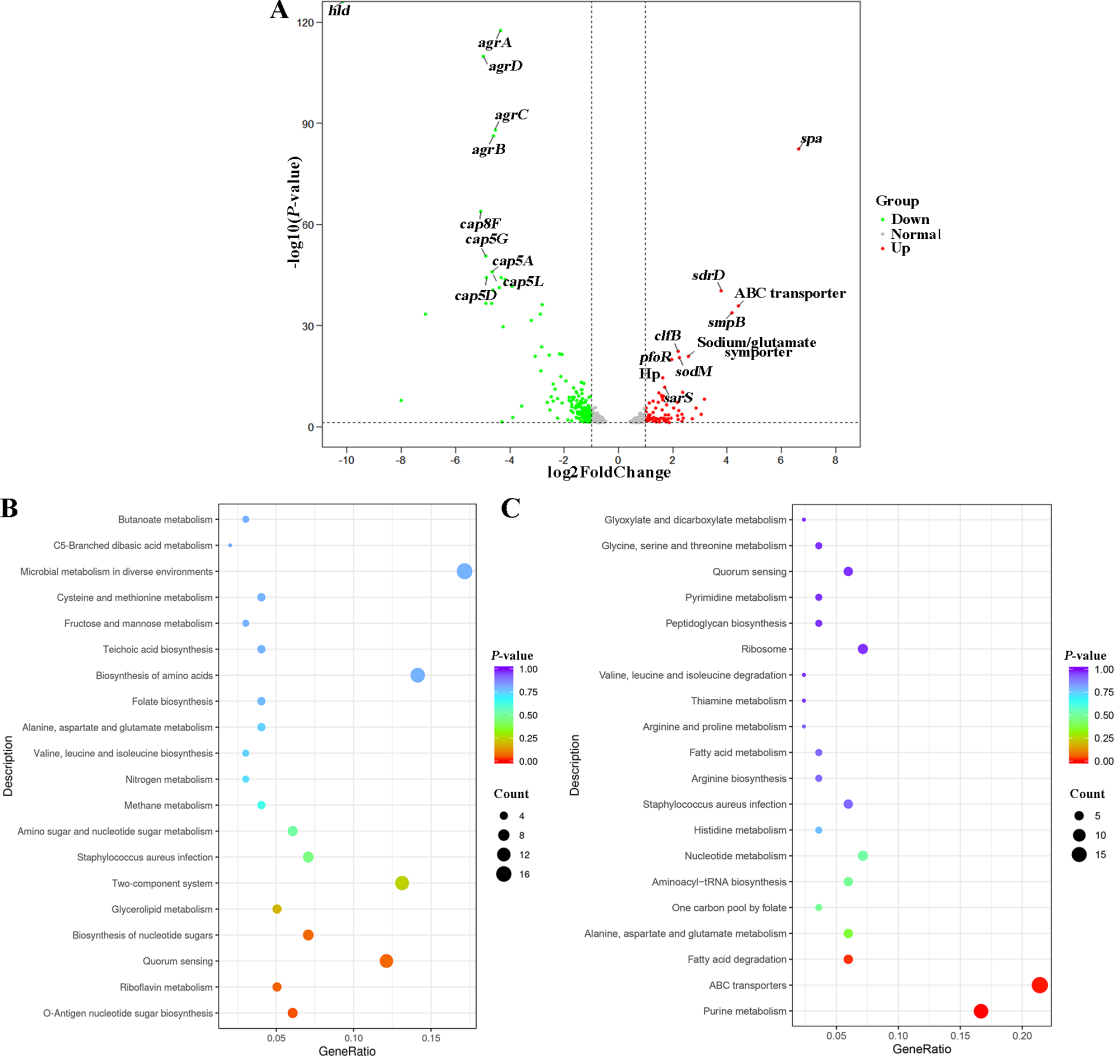
Transcriptome sequencing analysis in strains 23B and 23H. (A) The differentially expressed genes in strain 23B compared to strain 23H. Red group represented upregulated genes, green group represented upregulated genes, and gray group represented genes that no significant change in expression. (B) Identification of the top 20 upregulated pathways through enrichment analysis of differentially expressed genes with ascending *P*-value. (C) Identification of the top 20 downregulated pathways through enrichment analysis of differentially expressed genes with ascending *P*-value.

Transcriptome sequencing analysis identified a total of 2,480 expressed genes, with 248 (10%) exhibiting differential regulation in strains 23B and 23H (Table S3). Among these, 75 (3.0%) genes were significantly upregulated, while 173 (7.0%) genes were distinctly downregulated, as depicted in Fig. 4A. The most significant observation was the drastic reduction in the expression level of the hemolysin gene *hld* in strain 23B, which decreased by approximately 1,000-fold compared to that in strain 23H (*P <* 0.01). Moreover, the expression levels of the four accessory gene regulators, *agrA*, *agrB*, *agrC*, and *agrD*, in strain 23B were significantly diminished by 20- to 30-folds (*P <* 0.01) compared to strain 23H. Additionally, the expression of five genes related to capsular polysaccharide biosynthesis, *cap5A*, *cap5D, cap8F*, c*ap5G*, and *cap5L*, was significantly downregulated in strain 23B compared to that in strain 23H. Conversely, the expression of surface protein-encoding genes, including *spa*, *clfB*, *sdrC,* and *sdrD*, which are categorized as microbial surface components recognizing adhesion matrix molecules (MSCRAMMs), was significantly increased in strain 23B (17) (Fig. 4A; Table S3). Interestingly, some genes previously linked to biofilm formation, such as polysaccharide intercellular adhesin (PIA) encoded by the *ica* operon (18), biofilm-associated protein (BAP) (19), and surface protein encoded by *sasG* (20), showed no significant expression differences between the two *S. aureus* strains. This suggests that the enhanced biofilm formation observed in strain 23B was primarily due to the increased expression of MSCRAMMs-related genes (21). Additionally, metabolic-related genes and enzymes, such as sodium/glutamate symporter, *sodM*, *pfoR*, along with four transporter or membrane-related genes and factor, ABC transporter, *smpB*, Hp, *sarS* in strain 23B, also exhibited significant increases in comparison to strain 23H (Fig. 4A). In addition to the 20 genes with significantly different expression levels between the two *S. aureus* strains mentioned earlier, an additional 228 differentially expressed genes encode proteins with a variety of functions, including virulence factors (e.g., superantigen-like protein and gamma-hemolysin H-gamma-II subunit) and oxidative stress-related proteins (e.g., proline/betaine transporter, superoxide dismutase, squalene desaturase, and squalene synthase) (22–24).

To validate the accuracy of the gene expression profiles obtained through RNA-seq, 10 differentially expressed genes representing diverse functions were selected for analysis using qRT-PCR. The findings demonstrated that the mRNA levels of these 10 genes, as measured by qRT-PCR, were basically consistent with the RNA-seq results (Table S5), affirming the general reliability of the RNA-seq data.

To further elucidate the functions of the differentially expressed genes, all such genes were mapped to the KEGG database for pathway enrichment analysis. The analysis revealed that a total of 63 pathways were enriched. Figure 4B and C present the top 20 pathways upregulated and downregulated in strain 23B compared to strain 23H, respectively, selected based on ascending *P*-values. The most significant pathways upregulated predominantly include those related to metabolism, such as purine metabolism (14 genes), fatty acid degradation (5 genes), alanine, aspartate, and glutamate metabolism (5 genes), and aminoacyl-tRNA biosynthesis (5 genes). Additionally, 4 enrichment pathways exhibited upregulation: ABC transporters (18 genes), *Staphylococcus aureus* infection (5 genes), quorum sensing (5 genes), and ribosome (6 genes). On the contrary, downregulation was observed in pathways such as O-antigen nucleotide sugar biosynthesis (6 genes), quorum sensing (12 genes), two-component system (13 genes), *Staphylococcus aureus* infection (7 genes), and other metabolism-related pathways.

### The results of ATP assays

Given the association of persister formation between *S. aureus* with ATP depletion (25), we evaluated the ATP levels in exponential and stationary phase populations of strains 23B and 23H. Our findings confirmed a significant increase in ATP levels in strain 23B during the exponential phase (1.26-fold, *P* < 0.01), and significant reduction during the stationary phase (0.44 fold, *P* < 0.01) compared to that of strain 23H (Fig. 3C).

## DISCUSSION

Once *S. aureus* becomes firmly established in the infected tissue, certain regulatory traits may be fixed by spontaneous mutations occurring during chronic infection, likely due to selection pressure exerted by a vast number of yet undefined host factors (26). The *agr* system, a key regulator in *S. aureus*, plays a pivotal role in regulating virulence and adhesion factors critical for the pathogen’s lifecycle and interaction with the host. However, the genetic sequence of the four-gene *agr* operon lacks stability, frequently experiencing loss-of-function mutations that lead to nonfunctional *agr* system activity. An analysis conducted by Vishnu Raghuram et al. on over 40,000 *S. aureus* genomes revealed that more than 5% of these genomes exhibited frameshift mutations in the *agr* system, with the *agrC* gene showing the highest variety of such mutations (27). It has also been reported that 9% of healthy human subjects were colonized with *agr*-defective strains (28), and 22% of *S. aureus* isolates from chronically infected patients with atopic dermatitis displayed an *agr* mutant-like phenotype (29). These observations of *agr* system frameshift mutations in *S. aureus* suggest that their significance extends far beyond mere coincidence, potentially revealing crucial evolutionary implications for various strains of this pathogen. The *agr* system of *S. aureus* exemplifies the complex interplay between genetic evolution and environmental adaptation, encompassing interactions with host organisms. This dynamic interaction ultimately shapes the evolution of *S. aureus* strains, underscoring the multifaceted nature of microbial evolution and adaptation. In this study, two *S. aureus* strains were isolated from a single patient suffering from chronic inflammation. Notably, one strain displayed a complete hemolytic ring, while the other, due to an *agrC* frameshift mutation, exhibited an incomplete hemolytic phenotype. Although the mutated strain 23B exhibits reduced virulence compared to strain 23H, our *in vitro* experimental analyses demonstrated that 23B displayed enhanced adherence to A549 cells compared to its counterpart, 23 h, and exhibited superior biofilm formation capabilities. Transcriptomic data further indicated a significant upregulation in the expression of genes encoding surface proteins, including *spa*, *clfB*, and *sdrD*, in strain 23B. These findings suggest that under the pressure of various stressors, particularly in patients with chronic infections, *S. aureus* may undergo *agr* system mutations to enhance its survival and adaptability within the host.

Apart from enhancing adhesion and biofilm formation, bacterial strains present in infected patients frequently undergo mutations that confer resistance to antimicrobial agents due to selective pressures. Alterations in the susceptibility phenotypes of bacterial strains enable the straightforward detection of such mutations within clinical environments. However, bacterial strains may develop antibiotic tolerance, which is more elusive and often goes undetected in standard clinical testing, yet it can also lead to the failure of clinical treatments (30). In this study, despite the occurrence of an *agrC* frameshift mutation in strain 23B, no difference was observed in the MICs of antimicrobial agents against strains 23B and 23H. Both strains 23B and 23H exhibited susceptibility to nearly all antimicrobial agents, including vancomycin and daptomycin. However, time-kill curve analyses revealed that strain 23B demonstrated significantly higher survival rates than strain 23H following vancomycin treatment at 2, 4, 8, and 10 h. Previous literature has revealed that defective *agr* function may provide phenotypes selected in a pleiotropic manner, including reduced susceptibility to vancomycin (31). Similarly, although daptomycin MICs did not differ between strains 23B and 23H, strain 23B exhibited enhanced survival at 8 and 10 h of daptomycin exposure. Carlos M. Suligoy et al. similarly observed in isogenic *S. aureus* strain pairs, one with *agr* wild-type and the other *agr*-defective, that the MIC values for daptomycin remained unchanged, while significant differences existed in the time-kill curves (32). Interestingly, no significant differences were observed in the time-kill curve between strains 23B and 23H for the other three antimicrobial agents, including levofloxacin, gentamicin, and oxacillin. Given that both vancomycin and daptomycin belong to the class of glycopeptide antibiotics, the potential mechanism of tolerance to these antimicrobial agents in the strains may be related to the loss of *agr* system function and the consequent reduction in PSMs synthesis leading to altered lipid secretion. However, further research is necessary to substantiate this hypothesis.

In addition, previous study showed the crucial role of the *agr* system in the control of catabolic pathways, nutrient uptake, and energy metabolism (11). Transcriptional analysis of strains 23B and 23H indicated that the enrichment pathways of differentially expressed genes were predominantly related to metabolism. Notably, genes associated with purine metabolism garnered attention, with 14 such genes showing upregulation in strain 23B compared to strain 23H. Previous research suggested an increased consumption of ATP by genes involved in purine metabolism within *S. aureus* (33). Furthermore, compared to strain 23H, strain 23B exhibited significant upregulation of ABC transporters, with 18 genes showing increased expression (34). Measurement of ATP content in strains revealed higher ATP levels in strain 23B during the exponential phase. However, during the stationary phase, the ATP content of strain 23B declined rapidly and was markedly lower than that of strain 23H. Prior research has linked the formation of persistence in *S. aureus* to ATP consumption (25). These results suggest that dysfunction of *agr* system could potentially enhance the retention of *S. aureus* within the host by decreasing ATP levels, possibly through the regulation of metabolism and ABC transporters.

In conclusion, our research underscores that frameshift mutations in *agrC* significantly bolster the capacity of *S. aureus* to adapt and endure within the host during prolonged infections, manifesting through increased adhesion and biofilm formation capabilities, the development of tolerance to antimicrobial agents, and a decrease in ATP levels. Although these mutations result in diminished virulence, the consequent reduced toxicity might not necessarily be a disadvantage. In fact, in the context of chronic ailments such as cystic fibrosis, bacteremia, and osteomyelitis, this diminished virulence could potentially offer a survival advantage to the pathogen (27). This supports the hypothesis that a non-functional *agr* system may benefit *S. aureus* by promoting a phenotype better adapted for survival within the host. Consequently, it is of clinical significance to monitor these mutations in *S. aureus*, as they may present challenges in treatment strategies.

## MATERIALS AND METHODS

### Case description, bacterial strains, and antimicrobial susceptibility testing

A 65-year-old emergency patient, was diagnosed with post-thoracolumbar spine surgery infection and admitted to the hospital on 30 August 2023. Upon admission, the patient’s C-reactive protein (CRP) level was significantly elevated to 232.8 mg/L, and the patient’s procalcitonin (PCT) level remained at normal levels from admission to discharge. Consequently, the patient received treatment with vancomycin and imipenem, resulting in a reduction in CRP levels. On September 3rd, two *S. aureus* strains were simultaneously isolated from the patient’s blood samples; one displayed a complete hemolytic ring (β-hemolytic phenotype, designated as 23H), while the other showed an incomplete hemolytic phenotype (SIHP, designated as 23B). From September 5th to September 19th, the patient received a combination therapy of levofloxacin, cefmetazole, and vancomycin. Subsequently, vancomycin treatment was continued from September 20th to October 9th. However, the patient’s CRP levels began to rise on September 27th, peaking on September 28th, prompting consideration of a possible infection (Fig. 1). After debridement of the patient’s primary infectious focus, the patient’s CRP levels rapidly decreased until reaching a normal level. Nevertheless, no relevant specimens were sent for testing, and consequently, no bacteria, including *S. aureus*, were detected. The antimicrobial susceptibilities of the *S. aureus* strains were evaluated using the broth microdilution method in accordance with the standards set by the Clinical and Laboratory Standards Institute (CLSI) (35). The susceptibilities of the tested antimicrobial agents were interpreted following the current CLSI guidelines (36).

### Transmission electron microscope observation

Using a sterile toothpick, pick several fresh bacterial colonies and suspend them in phosphate buffer saline (PBS). Subsequently, adsorb the samples onto a carbon-coated grid, stain with 1% phosphotungstic acid (PTA, pH 6.8), and observe under a transmission electron microscope (Tecnai12, FEI, USA).

### Analysis of hemolytic capacity

Bacterial cultures were incubated in Brain-Heart Infusion broth (BHI, Oxoid, Massachusetts, USA) at 37℃with a shaker. After incubation for 4 h, the cultures were centrifuged at 100,000 × *g* for 8 min to collect the supernatants. Then, 100 µL of the supernatants was combined with 900 µL of a 1% fresh rabbit red blood cell suspension and incubated at 37℃ for 3 h. Each bacterial strain was tested in triplicate. The fresh culture medium without bacteria served as the negative control, while distilled water was used as the positive control. The level of hemolysis was determined by the formula: (OD540 of the sample − OD540 of the negative control)/(OD540 of the positive control − OD540 of the negative control) × 100%.

### Whole-genome sequencing and analysis

Genomic DNA of two *S. aureus* strains was subjected to draft-genome sequencing using a paired-end library on Illumina NovaSeq 6000 system. After being removed low-quality sequences and adapters, the reads were *de novo* assembled by the SPAdes Genome Assembler using multiple k-mer sizes (21, 33, 55, 77, 99, 127) and the “careful” option enabled for improved accuracy (v3.11.1) (37). Sequence and Spa typing were identified at CGE (https://cge. cbs.dtu.dk/services/) using the MLST (v2.0) (38) and SpaFinder (v1.0). The annotation of the strains was carried out using the Prokka (v1.14.6) (39) and Snippy (v4.6.0) (freely available on GitHub) was performed to compare the genome differences among two strains.

### Macrophage survival assay

To conduct the macrophage survival assay, murine macrophage RAW264.7 cells (1 × 10^5^ cells/well) were seeded in 12-well plates and incubated with a multiplicity of infection (MOI) of 100:1 of bacteria for 4 h. To eliminate extracellular bacteria, gentamicin and vancomycin were added at a concentration of 500 µg/mL. The drug-containing medium was removed, and the cells were washed with 1 mL of bacteria-free PBS by gentle shaking along the walls. The cells were lysed with 0.1% saponin (Sigma, Missouri, USA) for 20 min. To enumerate surviving bacteria, the mixture in 12-well plates was plated on blood agar plates. The experiment was repeated independently three times.

### Adhesion assay

Following a procedure similar to the macrophage survival assay, human alveolar epithelial A549 cells were seeded in 12-well plates (1 × 10^5^ cells/well) and then exposed to bacteria at a MOI of 100:1 of bacteria for 3 h. Non-adherent bacteria were removed by washing with PBS. Subsequently, cells were lysed with 300 µL of 0.1% saponin for 10 min. The lysates were gently shaken to ensure thorough mixing and then plated onto blood agar plates to quantify the number of adherent bacteria. The experiment was repeated independently three times.

### Virulence testing in the *Galleria mellonella* infection model

Overnight bacterial cultures were diluted in normal saline to obtain a concentration of 1 × 10^8^ CFU/mL. Wax moth larvae weighing between 250 and 300 mg (Tianjin Huiyude Biotech Company, Tianjin, China) were injected with 10 µL bacterial suspension and subsequently incubated at 37℃ for 72 h. The survival rates of *G. mellonella* were meticulously recorded at intervals of 12, 24, 36, 48, and 72 h, respectively. Normal saline was used as the negative control, respectively. Each isolate was tested in 10 larvae, and all experiments were done in triplicate. Kaplan-Meier survival curves were plotted using GraphPad Prism.

### Virulence assess by cell viability assay

The cell viability assay was conducted by quantifying the activity of lactate dehydrogenase (LDH) released from damaged cells using the LDH cytotoxicity assay kit (MedChemExpress, New Jersey, USA). A549 cells were seeded in 96-well plates (1 × 10^4^ cells/well) for 24 h. The culture solution was discarded, and 100 µL of bacterial dilution liquid (1 × 10^8^ CFU/ml) was added to each well. After 24 h of incubation, the plate was centrifuged at 3,000 × *g* for 5 min, and 50 µL supernatant of each well was transferred to a new 96-well plate. In each well, 50 µL of working solution was added and mixed, followed by incubation at 37℃ in the dark for 30 min, and then 50 µL of stop solution was added. Cell-free culture medium served as blank control, and medium-treated cell-seeded well served as negative control. Cells lysed with LDH-releasing reagent were used as a positive control, and cell-free medium with LDH-releasing reagent served as high background control. Cytotoxicity was calculated as follows: (OD490 of sample-OD490 of negative control)/[(OD490 of positive control−OD490 of high background control)−(OD490 of negative control−OD490 of blank control)]×100%. All experiments were performed in triplicates.

### Time-killing assay

Overnight bacterial cultures were diluted in BHI broth to obtain a concentration of 10^8^ CFU/mL. The bacterial suspension was then treated with daptomycin (40 µg/mL), levofloxacin (4 µg/mL), vancomycin (10 µg/mL), oxacillin (1.5 µg/mL), or gentamicin (8 µg/mL) for 24 h. At time intervals (0, 2, 4, 8, 10, and 24 h), an aliquot of cells was removed, diluted in BHI broth, and plated to enumerate survivors. All experiments were performed in triplicates.

### Biofilm formation

To cultivate biofilms, 100 µL of overnight bacterial cultures, standardized to an OD600 of 0.1 with BHI, was introduced into the wells of a 96-well plate. After incubating for 48 h, and the wells were washed with 150 µL of sterile water. Subsequently, 160 µL of 0.1% crystal violet solution was added to each well, followed by a 30 min incubation at 25℃. Subsequently, the wells were washed thrice with 150 µL of sterile water. Afterward, 180 µL of a 75% ethanol solution was added to each well to dissolve the crystal violet dye. After a 30 min incubation at 37℃, the absorbance of each well was measured at 595 nm using Epoch 2 Microplate Spectrophotometer (BioTek Instruments). All experiments were performed in triplicates.

### Growth curves

The overnight bacterial cultures were diluted to a concentration of 1 × 10^6^ CFU/mL. Following this, the diluted suspension was incubated for 24 h at 37℃ within a 96-well plate. To monitor growth, the absorbance at 600 nm was measured at 30 min intervals utilizing an Epoch 2 Microplate Spectrophotometer. All experiments were performed in triplicates.

### RNA-Seq and reads mapping

RNA-Seq analyses were carried out using three independent RNA samples from each bacterial strain. Complementary DNA (cDNA) from the RNA samples was sequenced on an Illumina Novaseq platform and 150 bp paired-end reads were generated. After being removed low quality and reads adapter, the reads were aligned and mapped to the reference genome of *S. aureus* (accession number: NC_007795.1) using Bowtie2 (v2.2.3) (40). HTseq (v0.9.1) (41) was used to count the number of reads mapped to each gene, and then FPKM of each gene was calculated based on the length of the gene and the count of reads mapped to this gene. Differential expression analysis of strains 23B and 23H was compared using the R package edgeR (version 3.24.3) (42), and correction of the *P*-value was performed by the Benjamini–Hochberg method. Corrected *P*-value of 0.05 and log2(Fold change) of 1 was set as the threshold for significantly differential expression. The gene ontology (GO) annotation and Kyoto Encyclopedia of Genes and Genomes (KEGG) pathway analysis of differentially expressed genes was implemented by the clusterProfiler package (version 3.8.1) (43).

### RT-qPCR experiments

Complementary DNA (cDNA) synthesis from total RNA extracted from bacterial cultures in the post-exponential growth phase was performed using the FastKing RT Kit reverse transcription system (TIANGEN, Beijing, China) according to the manufacturer’s instructions. The resulting cDNA was then amplified by qPCR using Taq Pro Universal SYBR qPCR Master Mix (Vazyme, Nanjing, China) on the LightCycler 480 II instrument (Roche, Basel, Switzerland) adhering to the manufacturer’s protocol. The primers used in this study were listed in Table S4. All quantitative RT-PCR experiments were performed in duplicate, with *gyrB* as an internal control (44).

### ATP assays

ATP levels of stationary (4 h) and exponential (8 h) bacterial cultures were measured using a Promega BacTiter Glo kit (Promega, Madison, USA) according to the manufacturer’s instructions.

### Statistical analysis

The Mann-Whitney *U* rank sum test was applied to comparisons of related data between two *S. aureus* strains. *P-*values < 0.05 were considered statistically significant. All the statistical analyses were performed by Statistical Package for the Social Sciences (SPSS, v24.0).

## Data Availability

All assembled sequence data have been deposited in GenBank under the BioProject accession number PRJNA1088786.

## SUPPLEMENTAL MATERIAL

Supplemental material spectrum.01497-24-s0001.xlsx Tables S1 to S5.

ASM does not own the copyrights to Supplemental Material that may be linked to, or accessed through, an article. The authors have granted ASM a non-exclusive, world-wide license to publish the Supplemental Material files. Please contact the corresponding author directly for reuse.
